# Extending effective multiplicity for independent designs in multichannel fNIRS studies

**DOI:** 10.1117/1.NPh.13.3.035005

**Published:** 2026-08-12

**Authors:** Yuki Yamamoto, Wakana Kawai, Tatsuya Hayashi, Yuki Ishikawa, Minako Uga, Yasushi Kyutoku, Ippeita Dan

**Affiliations:** a Chuo University, Faculty of Science and Engineering, Tokyo, Japan; b Ibaraki University, Ibaraki, Japan; c Health Science University, Department of Welfare and Psychology, Faculty of Health Science, Yamanashi, Japan; d Chuo University, Faculty of Global Management, Tokyo, Japan

**Keywords:** functional near-infrared spectroscopy, multiple comparison, family-wise error rate, false discovery rate, *M*_eff_

## Abstract

**Significance:**

Recent advancements in multichannel functional near-infrared spectroscopy have led to an expansion in the number of measurement channels. This increase in multiplicity necessitates appropriate multiple-comparison correction. The effective multiplicity ($M_{\mathrm{eff}}$) method has been introduced to control family-wise error rate while accounting for correlation between channels. However, the application of this method has been limited to a one-sample design, and its performance has only been compared with Bonferroni correction.

**Aim:**

We aimed to evaluate the applicability of the $M_{\mathrm{eff}}$ method to independent designs in channel-wise analysis and to compare its performance with other conventional multiple-comparison correction methods.

**Approach:**

Using simulated datasets, we conducted resampling simulations to examine the relationship between the number of channels and $M_{\mathrm{eff}}$ values and between the number of channels and the number of significant channels for each correction method. In addition, we performed exploratory analyses using actual experimental datasets with 44-channel measurements from ∼60 participants, which had been analyzed within regions of interest in previous studies.

**Results:**

In resampling simulations, as the number of channels increased, $M_{\mathrm{eff}}$ values converged at around 25 for the 44-channel datasets, and the number of significant channels remained relatively constant regardless of the number of total channels, in contrast to other correction methods. In the exploratory analyses on actual experimental datasets, channels identified as significant were similar to the regions of interest defined in the previous studies.

**Conclusions:**

We demonstrated that, in channel-wise analysis, when the total number of participants exceeds the number of channels, the $M_{\mathrm{eff}}$ approach is effective for balancing type I and II errors in independent designs and that it is applicable to both exploratory and confirmatory analyses.

## 1 Introduction

Functional near-infrared spectroscopy (fNIRS) has been widely used as a noninvasive and convenient neuroimaging tool throughout the past three decades.^1^^–^^4^ Advances in fNIRS hardware and software have significantly improved its spatial resolution through multichannelization. Originally, fNIRS measurements were conducted using a single-channel system. The introduction of a frequency-division multiplexing system overcame the problem of optical interference between adjacent channels, thereby enabling the simultaneous monitoring of multiple brain regions.^5^ Currently, multichannel fNIRS systems with dozens of emitters and detectors are widely used in experimental studies.^3^

However, improvements in the spatial resolution of fNIRS through multichannelization have necessitated addressing the multiple comparisons problem in statistical hypothesis testing. When analyses were restricted to a single channel or region of interest (ROI) based on a prior hypothesis, the number of hypotheses to be tested was limited, and thus, the multiple-comparison problem was not a major concern. By contrast, when statistical hypothesis testing is performed across multiple channels, the number of null hypotheses corresponds to the number of channels. As each test is typically performed at a significance level of 5%, the overall risk of type I errors (false positives), in which nonactivated channels are identified as activated, increases with the number of channels.

To address the multiple comparisons problem, two representative approaches are to control the family-wise error rate (FWER) and the false discovery rate (FDR). The FWER refers to the probability of incorrectly rejecting at least one null hypothesis. One major conventional FWER control method is the Bonferroni correction.^6^ Bonferroni correction is the simplest and most conservative approach, in which the significance level is divided by the number of null hypotheses. On the other hand, the FDR control is designed to control the proportion of false positives among all rejected hypotheses. In multichannel analysis, the Benjamini–Hochberg (BH) procedure is widely applied as it provides higher power than the Bonferroni correction.^7^ FWER and FDR control methods typically assume that multiplicity equals the number of channels and that all tests are independent. However, fNIRS channels are often correlated, and therefore, this assumption does not hold. Consequently, conventional FWER control methods tend to be more conservative than theoretically expected significance levels, whereas FDR control may fail to adequately control for false positives.

One FWER correction method taking the correlation between channels into account is the resampling-based approach, such as permutation tests.^6^^–^^8^ The resampling-based approach is a nonparametric method that does not assume distribution shape and that uses the data itself to construct an empirical null hypothesis distribution.^6^ In FWER control, corrected p-values are calculated based on a null distribution generated by extracting the minimum p-value (Min P) or maximum t-value (Max T) across tests at each iteration. The permutation and bootstrap test based on the Max T distribution is applied to channel-wise analysis.^6^ Although this method provides a useful framework for accounting for correlation between channels in FWER correction, the application is not without limitations. In particular, as some significant activations may be contained in the maximum null distribution, this procedure may reduce statistical power.

Another promising FWER control method in fNIRS analysis is the effective multiplicity ($M_{\mathrm{eff}}$) method.^8^^,^^9^ This method was originally proposed as a multiple-comparison correction method in the field of genetics for genome-wide association studies.^10^^,^^11^ Genetics, like neuroimaging, has encountered challenges with type I errors due to a sudden explosion in available data, as millions of genetic variants are tested for correlations with specific outcomes. In this approach, the number of null hypotheses is estimated as $M_{\mathrm{eff}}$ using eigenvalues of the correlation matrix between the variables being tested. This value is then used to correct the statistical significance threshold for multiple comparisons. The application of the $M_{\mathrm{eff}}$ method for multiple testing among nonindependent variables is not limited to genetics as it can be applied to both small and large sets of variables, regardless of the degrees of intercorrelation.^12^ The formula for calculating the $M_{\mathrm{eff}}$ value has been further refined and expanded by several researchers,^13^^–^^17^ and in fNIRS studies, Galwey’s function has been used because it is optimized for multiple signals with strong correlations and can be handled continuously.^9^^,^^18^ Uga et al.^9^ applied the $M_{\mathrm{eff}}$ method to three sets of experimental data with different activation patterns and demonstrated through resampling simulations that the number of null hypotheses in 44-channel datasets were reduced from 44 to between 10 to 15. However, when the sample size is smaller than the number of channels, rank deficiency in the eigenvalue vector can lead to underestimation of $M_{\mathrm{eff}}$ values. To address this issue, an exponential model has been proposed for estimating reasonable $M_{\mathrm{eff}}$ values for datasets with a smaller sample size than the number of channels.^18^

Nonetheless, the application of the $M_{\mathrm{eff}}$ method to fNIRS analysis has been limited to one-sample t-tests for assessing significant activation or differences between conditions in paired samples. For paired t-tests, the $M_{\mathrm{eff}}$ value can be calculated from the difference between datasets, enabling the analysis to be treated as a one-sample t-test. However, for independent samples, it has not been possible to compute the difference from summary data. In such cases, a predefined ROI and FWER control with Bonferroni correction are still commonly used.^19^^–^^22^ Thus, the $M_{\mathrm{eff}}$ method should be further extended to be applicable to independent statistical designs, such as independent t-tests and ANOVAs, which are often employed in fNIRS experimental designs.

Hence, the objective of this study was to evaluate the applicability of the $M_{\mathrm{eff}}$ method to independent t-tests and ANOVA in fNIRS channel-wise analysis. In addition, we aimed to compare the $M_{\mathrm{eff}}$ method with other representative multiple-comparison correction methods and to provide a comprehensive discussion of dealing with the multiple testing problem in fNIRS channel-wise analysis. Three sets of experimental data from different experimental designs were selected from previously published studies. Based on these datasets, dummy datasets were created, and resampling simulations were conducted to compare the $M_{\mathrm{eff}}$ method with other correction methods. Independent t-test and ANOVA with the $M_{\mathrm{eff}}$ method were performed on the experimental data to verify whether the results of previous confirmatory studies could be reproduced. Based on these results, we discuss the validity of the $M_{\mathrm{eff}}$ method and representative correction methods for each independent statistical test.

## 2 Method

### 2.1 Correction Methods

In this study, we compared FWER control using $M_{\mathrm{eff}}$ with representative multiple-comparison correction methods. The Bonferroni correction, FDR method (BH), and the permutation method^6^ were used as comparative correction methods in this study. The results without applying any correction methods (no correction) were also included in the comparison. In the permutation method, permutation of the data was performed 1000 times to generate a Max T distribution for independent t-tests.^6^ Permutations were performed at the participant level by randomly reallocating participants to the two groups while preserving the original group sizes, relying on the exchangeability assumption that, under the null hypothesis, participants are interchangeable between groups. Similarly, 1000 iterations were performed to generate max F distributions for one-way ANOVAs. P-values for each channel (hereinafter CH) were calculated from these distributions.

Here, we describe the theoretical framework of the $M_{\mathrm{eff}}$ method employed in this study, using a typical fNIRS data structure as an example. For multichannel fNIRS data obtained from M CHs and N participants, summary data used for second-level analysis are represented as $\beta^{N\times M}$. The eigenvalue vector ($\lambda_{i}$) is then derived from the correlation matrix (M×M) as follows:

$$\lambda_{1},\lambda_{2},\ldots \lambda_{M}.$$ (1)

In this study, we applied Galwey’s function to calculate $M_{\mathrm{eff}}$ values, following previous research.^9^^,^^16^^,^^18^ As this function estimates the effective number of independent tests, the same function should be applied regardless of whether the statistical design is paired or independent.

$$M_{\mathrm{eff}}={\left(\overset{M}{\underset{i=1}{\sum }}\sqrt{\lambda_{i}}\right)}^{2}/\overset{M}{\underset{i=1}{\sum }}\lambda_{i}.$$ (2)

**Fig. 1:**
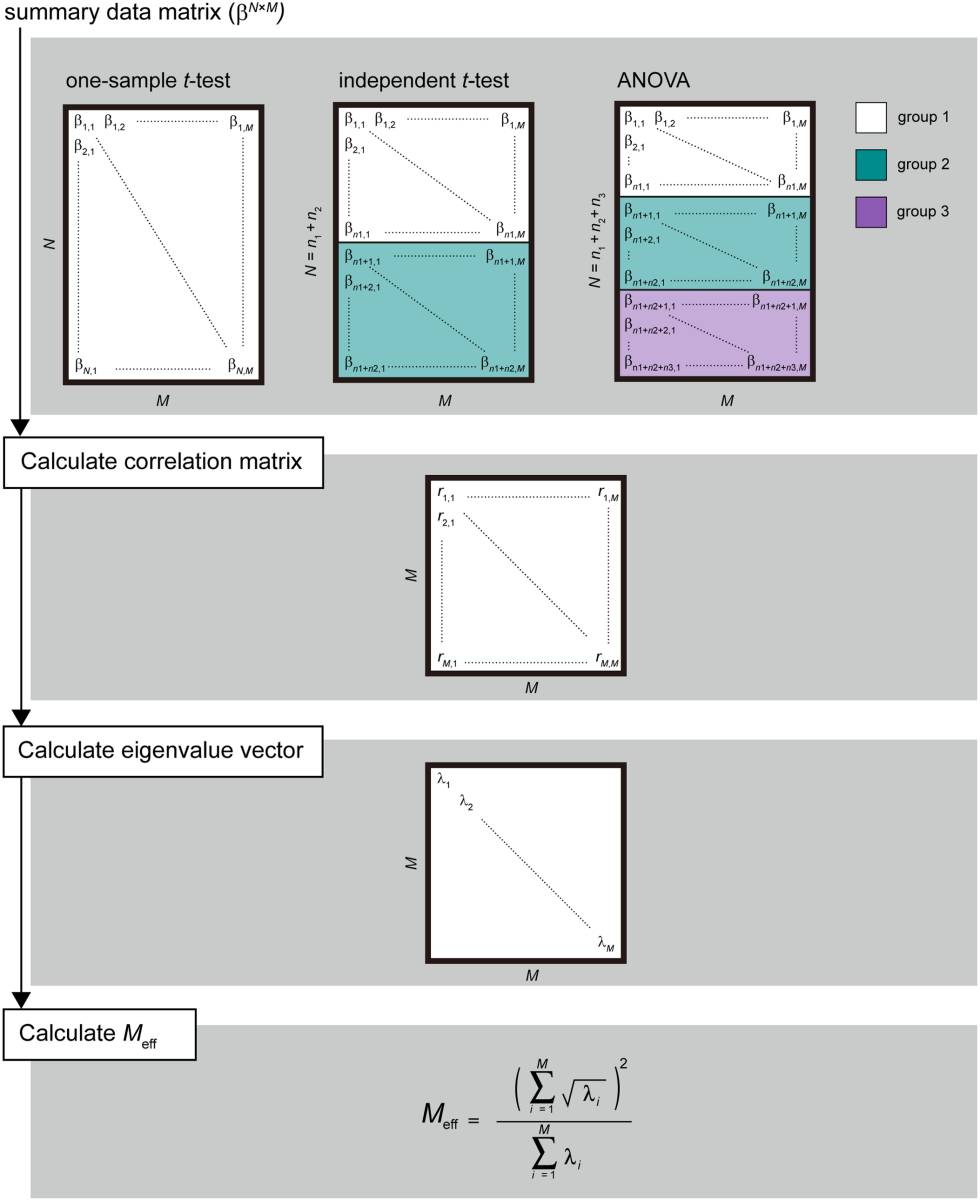
Calculation of $M_{\mathrm{eff}}$. The summary data matrix of each experimental dataset is first used to calculate a unique correlation matrix. Then, the correlation matrix is used to calculate an eigenvector. Finally, the $M_{\mathrm{eff}}$ value is calculated from the eigenvalues utilizing Galwey’s function.

In cases where fNIRS data are obtained from M CHs and k groups (with the number of participants in each group represented as $N_{1},N_{2},\ldots ,N_{k}$), the summary data for group analysis are represented as $\beta^{(\overset{k}{\underset{j=1}{\sum }}N_{i})\times M}$. The $M_{\mathrm{eff}}$ value for this data structure was calculated using the same procedure as described above (Fig. 1).

### 2.2 Experimental Procedure

In this study, we utilized three sets of experimental data obtained from separate studies.^21^^–^^23^ Below, we will describe the respective experimental procedures and participant profiles. Participants in Monden et al.^22^ included 30 right-handed typically developing (TD) children (20 boys and 10 girls, average age=9.7±2.3  years, age range 6 to 14) and 30 right-handed children with attention deficit hyperactivity disorder (ADHD) (25 boys and 5 girls, average age =9.1±2.6, age range 6 to 15). Participants in Tokuda et al.^23^ included 21 right-handed children with ADHD without co-occurring autism spectrum disorder (ASD) (17 boys and 4 girls, average age=7.8±1.7, age range 6 to 13) and 11 right-handed ADHD with ASD children (11 boys, average age=8.2±2.1, age range 7 to 14). Nagashima et al.^21^ included 22 right-handed TD children (15 boys and 7 girls, average age=9.8±2.0, age range 6 to 13) and 22 right-handed children with ADHD (19 boys and 3 girls, average age=9.5±2.0, age range 6 to 14).

Participants in both Monden et al.^22^ and Tokuda et al.^23^ performed a Go/No-go task requiring inhibitory control. The procedure consisted of six block sets, containing alternating go (baseline) blocks and no-go (target) blocks, each block lasting 24 s. In the go block, participants were presented with two different pictures in a random sequence and asked to respond to each picture by pressing a button as quickly as possible. In the Go/No-go block, participants were presented with two different pictures in a random sequence and asked to respond to one picture (50% of the trials; go trials) and inhibit their response to the other picture (no-go trials).

In Nagashima et al.,^21^ participants performed an oddball task. This task requires the detection of and response to infrequent target stimuli (oddballs) embedded within a sequence of repetitive trials. The procedure consisted of six block sets, alternating baseline and oddball blocks, each block lasting 25 s. In the baseline block, participants were presented with one picture and asked to press a blue button on a response box for that picture. In the oddball block, two different pictures were presented in sequence, and participants were instructed to press the blue button for the standard stimuli and the red button for the target (oddball) stimuli.

Written informed consent was obtained from the guardians of all participants in accordance with the latest version of the Declaration of Helsinki. These studies were, respectively, approved by the ethics committees of Jichi Medical Hospital and the International University of Health and Welfare. The obtained data were later irreversibly anonymized for secondary analyses.

In each experiment, fNIRS data were acquired from 44 CHs in total. Two 3×5 multichannel probe arrays, each consisting of eight light sources (emitters) and seven detectors arranged alternately, were used, yielding 22 CHs on each side of the participant’s head.

### 2.3 Evaluation of $M_{\mathrm{eff}}$ Using Simulated Data

#### 2.3.1 Generation of simulated data

To evaluate the effectiveness of the $M_{\mathrm{eff}}$ method for independent designs, resampling simulations were performed using simulated datasets generated from actual experimental data. The actual experimental data alone were not suitable for validating the proposed method, even through simulations, as only a small number of CHs exhibited significant differences with a large effect size in independent t-tests or ANOVAs. This reduces their suitability because, when only a few CHs have large effect sizes, whereas others have little to no effect size, it becomes difficult to compare the $M_{\mathrm{eff}}$ method with other correction methods because no significant differences can be expected to be detected regardless of the correction method. Thus, in this study, we generated simulated datasets with various effect sizes based on actual experimental data while maintaining their within-group correlation structure.

To evaluate the FWER controlling performance, we defined “truly significant channels” (TS CHs) in the simulated datasets. TS CHs in each dataset were randomly assigned with a reasonable effect size within a determined range. Reasonable effect sizes corresponding to the sample size of each dataset were determined using power analysis implemented in G*Power (version 3.1.9.7)^24^ and used as the threshold. We set four conditions for the number of TS CHs (5, 15, 25, 35) and generated 100 datasets for each condition.

Specifically, for the independent t-test, we used the TD and ADHD data from Monden et al.^22^ to generate simulated datasets. Power analysis was conducted under the following conditions: sample size of group 1: n=30; sample size of group 2: n=30; mean: difference between two independent means (two groups); and t-test with α=0.05, power=0.8, tail = two. Accordingly, Cohen’s d=0.74 was obtained as the minimum threshold of a reasonable effect size.

Based on the empirical effect size distribution observed in the actual experimental data, TS CHs were randomly assigned with effect sizes within the range of Cohen’s d=0.74 to 1.25, whereas the remaining CHs were randomly assigned with those within the range of −0.45 to 0.73. To assign the target effect size to each CH, we adjusted the mean difference between the two groups by adding fixed shifts (Δ) to the data of each CH in the TD group. This process allowed us to assign a specific effect size to each CH while preserving the empirical within-group correlation structure because within each CH, the same Δ was added to all data in the TD group. For each condition of the number of TS CHs, this procedure was repeated 100 times to generate 100 datasets.

For the one-way randomized-groups ANOVA (1 by 3, fixed-effects design), we used the TD data from Monden et al.^22^ and ADHD without ASD and ADHD with ASD data from Tokuda et al.^23^ to generate simulated datasets. Power analysis was conducted under the following conditions: sample size: n=62; ANOVA: fixed effects, omnibus, one-way; and F-test with α=0.05, power=0.8. As a result, $\eta^{2}=0.143$ was obtained as the minimum threshold for a reasonable effect size.

Based on the empirical effect size distribution in the actual experimental data, TS CHs were randomly assigned with effect sizes within the range of $\eta^{2}=0.143$ to 0.225, whereas the remaining CHs were randomly assigned within the range of 0 to 0.142. To assign the target effect size to each CH, we first calculated the within-group variance from the actual data. Then, we adjusted the group-mean according to the contrast [1,0,−1] to achieve the target $\eta^{2}$. Similar to the process in the independent t-test, this process allowed us to assign the target $\eta^{2}$ on each CH while maintaining the within-group correlation structure of the actual data because although this changed the group means, it did not alter deviations from the group means. For each condition of the number of TS CHs, this procedure was repeated 100 times to generate 100 datasets.

#### 2.3.2 Comparing the relationship between the number of CHs tested and the number of significant CHs across correction methods

Using the generated simulated datasets, we examined the relationship between the number of CHs tested and the number of significant CHs detected by the $M_{\mathrm{eff}}$ method. The predefined TS CHs were kept constant, and the remaining CHs were randomly added one by one to incrementally increase the total number of CHs. For each total CH count, the number of significant CHs corrected by the $M_{\mathrm{eff}}$ method was calculated. This procedure was applied to 100 datasets for each of the four conditions, and the average values and SD of the number of significant CHs, type I error rate, and statistical power were calculated across the 100 datasets. For comparison, this relationship was also examined for the uncorrected cases, the Bonferroni correction, the FDR method (BH), and the permutation method (Max T or Max F). The significance level was set at α=.05 for all statistical tests and correction methods, and t-tests for all correction methods were two-tailed.

### 2.4 Evaluation of $M_{\mathrm{eff}}$ Using Actual Experimental Data

#### 2.4.1 Exploration of the relationship between the number of CHs and $M_{\mathrm{eff}}$ by resampling simulation

To explore the relationship between the number of CHs and $M_{\mathrm{eff}}$, we randomly resampled a given number of CHs (M:1≦M≦44) for each dataset and calculated $M_{\mathrm{eff}}$. For each M, we randomly selected M CHs from the original 44-CH dataset. This resampling was performed 1000 times, and the average $M_{\mathrm{eff}}$ value and standard deviation (SD) were calculated for each M. For the independent t-test, we used the TD and ADHD data from Monden et al..^22^ For the one-way randomized-groups ANOVA, we used the TD data from Monden et al.^22^ and ADHD without ASD and ADHD with ASD data from Tokuda et al.^23^

#### 2.4.2 Application of the $M_{\mathrm{eff}}$ method to actual experimental data

To evaluate the validity of the proposed method, we conducted an exploratory analysis of experimental data using independent t-tests or one-way randomized-groups ANOVAs with the $M_{\mathrm{eff}}$ method, aiming to reproduce the results of previous studies. In Monden et al.,^22^ CH28 and CH32 (right CH6 and CH10) were set as the ROI and were reported to exhibit significant differences between the TD and ADHD groups. Similarly, we conducted an independent two-sample t-test with the $M_{\mathrm{eff}}$ method between the TD and ADHD groups across all CHs to confirm whether the result at the ROI could be reproduced. In Nagashima et al.,^21^ CH32 (right CH10) was set as the ROI and was reported to exhibit significant differences between the TD and ADHD post PLA groups (ADHD children after administration of a Placebo). Similarly, we conducted an independent two-sample t-test with the $M_{\mathrm{eff}}$ method between the TD and ADHD post PLA groups across all CHs to confirm whether the result at the ROI could be reproduced. For the independent one-way ANOVA, data from the TD group in Monden et al. ^22^ and the ADHD without ASD and ADHD with ASD groups in Tokuda et al.^23^ were used. Monden et al. ^22^ reported significant differences in CH28 and CH32 between the TD and ADHD groups. In this study, we conducted a one-way randomized-groups ANOVA with the $M_{\mathrm{eff}}$ method across the three groups to verify whether significant differences could be reproduced in the same CHs.

## 3 Results

### 3.1 Evaluation of $M_{\mathrm{eff}}$ Using Simulated Data

**Fig. 2:**
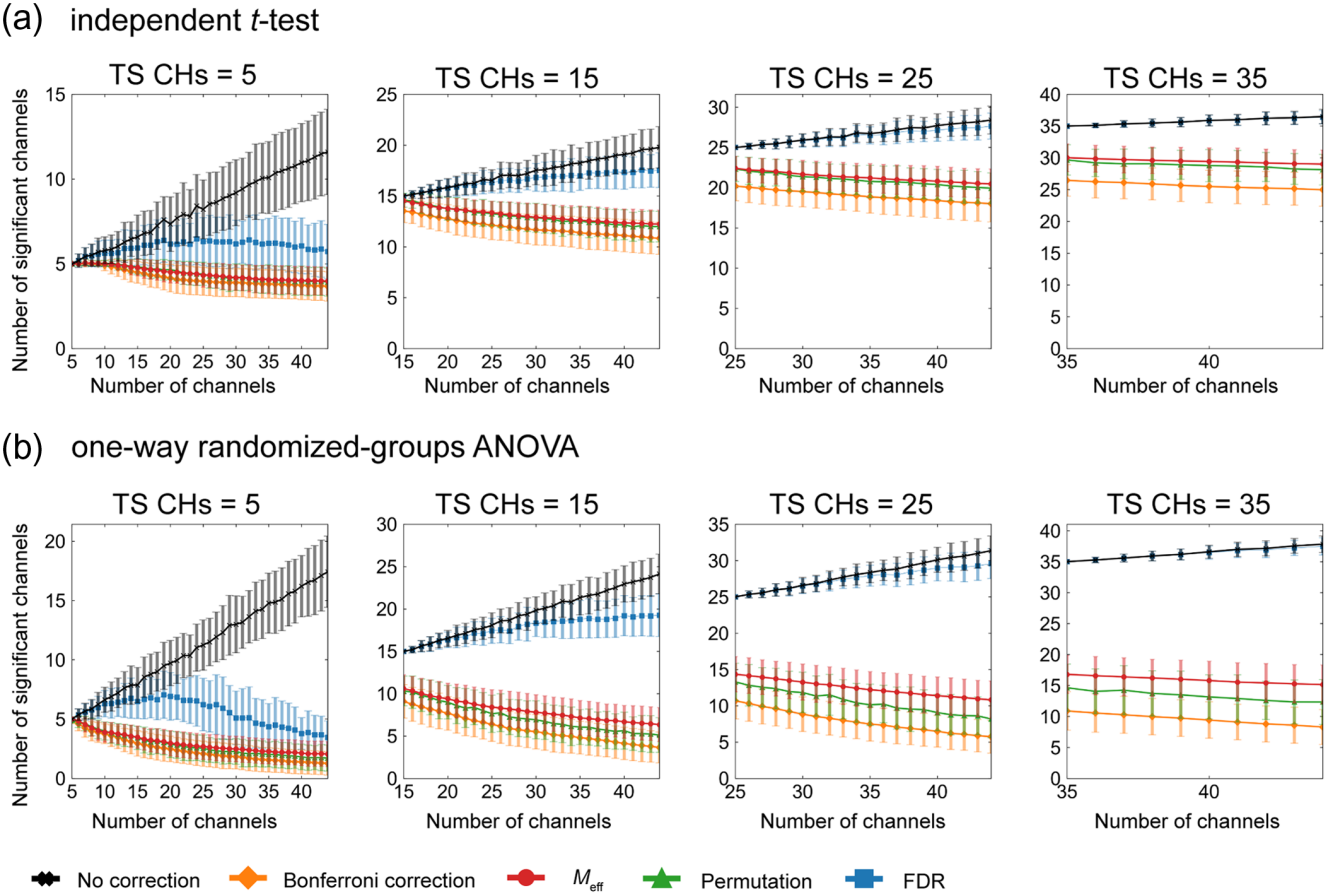
Comparison of correction methods in the relationship between the number of channels and the average number of significant channels for each TS CHs condition (TS CHs = 5, 15, 25, 35): (a) independent t-test, (b) one-way randomized-groups ANOVA. Error bars represent SD. TS CHs, truly significant channels.

**Fig. 3:**
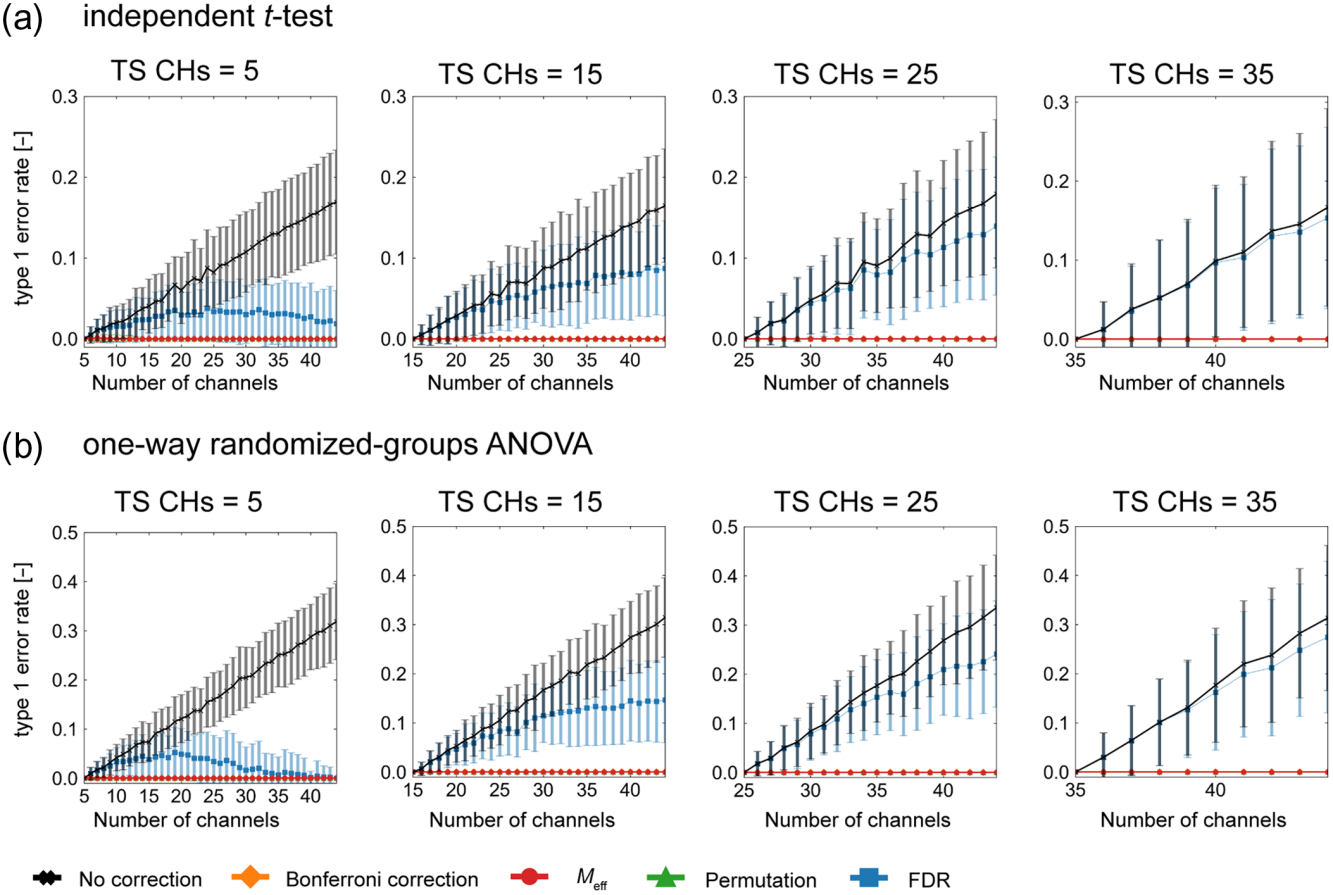
Comparison of correction methods in the relationship between the number of channels and the type 1 error rate for each TS CHs condition (TS CHs = 5, 15, 25, 35): (a) independent t-test, (b) one-way randomized-groups ANOVA. Error bars represent SD. TS CHs, truly significant channels.

**Fig. 4:**
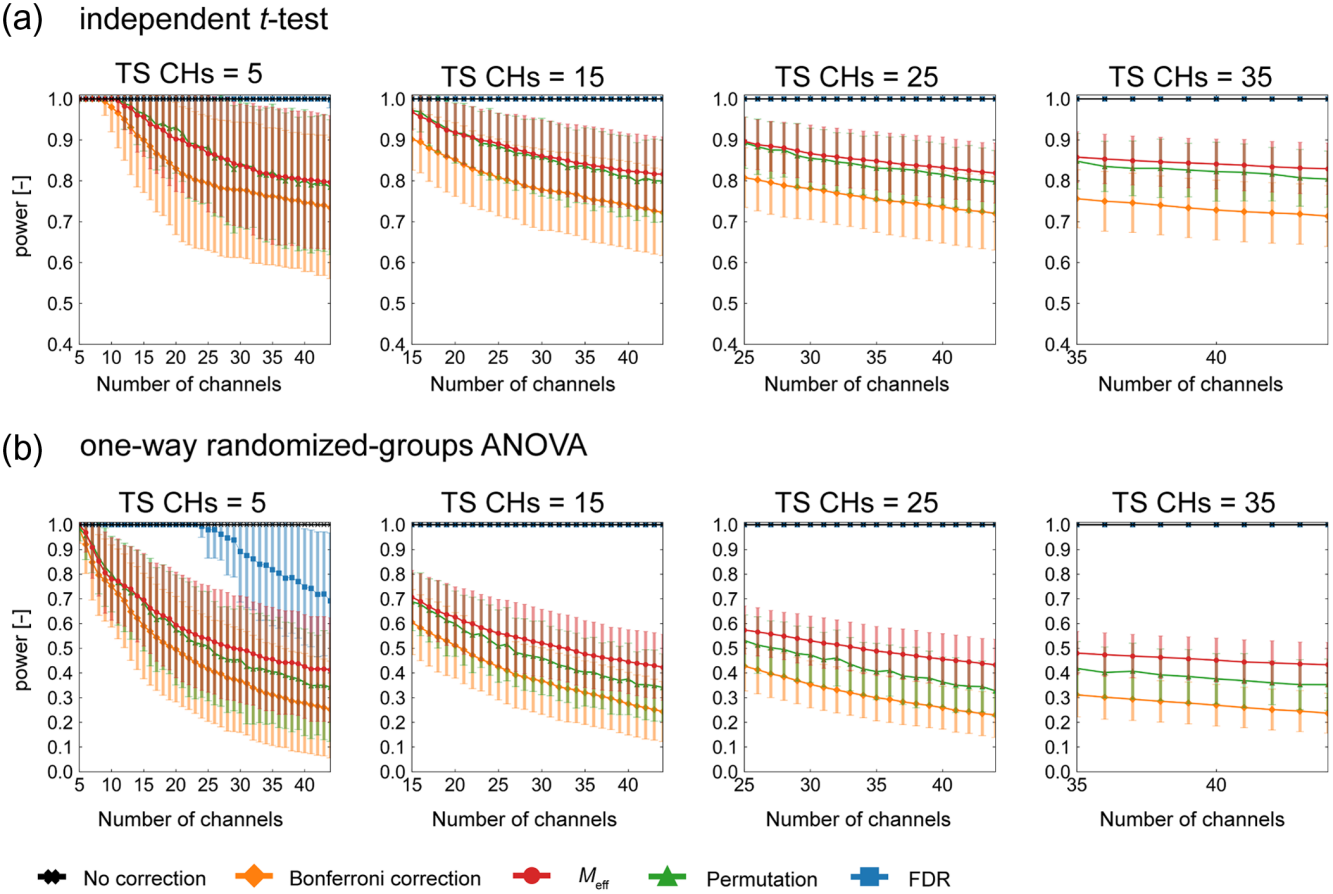
Comparison of correction methods in the relationship between the number of channels and power for each TS CHs condition (TS CHs = 5, 15, 25, 35): (a) independent t-test, (b) one-way randomized-groups ANOVA. Error bars represent SD. TS CHs, truly significant channels.

For each of four conditions (TS CHs = 5, 15, 25, 35), the number of significant CHs, type I error rate, and statistical power were calculated. These values were derived for each of the following cases: no correction, Bonferroni correction, FDR method (BH), permutation method, and $M_{\mathrm{eff}}$ method, and their mean and SD were plotted accordingly (Figs. 2–4). The number of significant CHs tended to be larger than the number of TS CHs for each condition in the cases of no correction and the FDR method. When controlling for FWER using Bonferroni correction, the permutation method, or the $M_{\mathrm{eff}}$ method, the number of detected significant CHs was less than the number of TS CHs and decreased as the number of CHs increased. The number of significant CHs detected using the $M_{\mathrm{eff}}$ method was larger than that detected by other FWER controlling methods, such as the Bonferroni and permutation methods.

### 3.2 Evaluation of $M_{\mathrm{eff}}$ Using Actual Experimental Data

#### 3.2.1 Exploration of the relationship between the number of CHs and the $M_{\mathrm{eff}}$ by resampling simulation

**Fig. 5:**
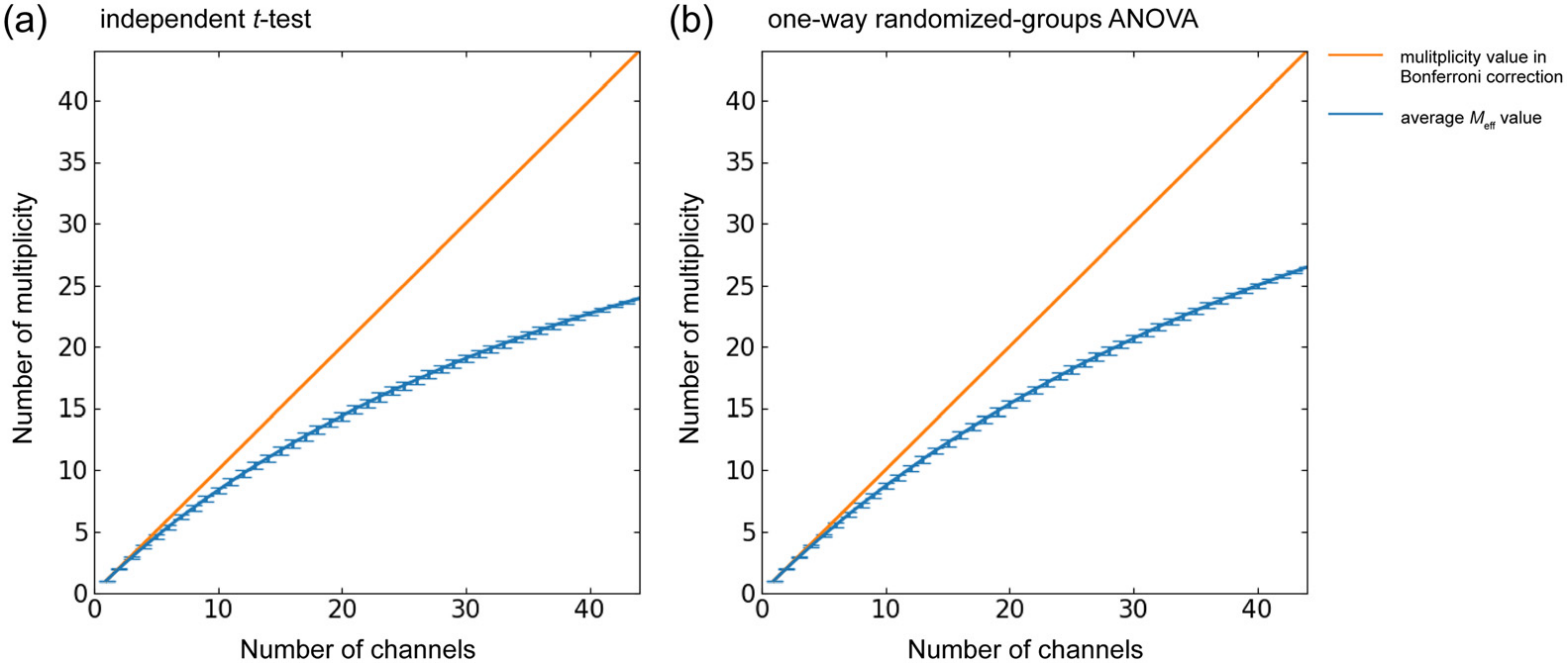
Relationship between number of channels and number of multiplicities: (a) independent t-test, (b) one-way randomized-groups ANOVA. Error bars for average $M_{\mathrm{eff}}$ values represent SD.

We plotted the average $M_{\mathrm{eff}}$ values and SD for each number of CHs for the dataset of independent t-tests and one-way randomized-groups ANOVAs (Fig. 5). Exact values are listed in Table S1 in the Supplementary Material. For comparison, we also plotted the multiplicity M values used in the Bonferroni correction. In the independent t-test, the increase in $M_{\mathrm{eff}}$ values was gradually controlled as the number of CHs increased, eventually converging to $M_{\mathrm{eff}}=23.94$ at CH=44. A similar trend was observed in the one-way randomized-groups ANOVA, where the $M_{\mathrm{eff}}$ eventually converged to $M_{\mathrm{eff}}=26.47$ at CH=44.

### 3.3 Application of $M_{\mathrm{eff}}$ to Actual Experimental Data

**Fig. 6:**
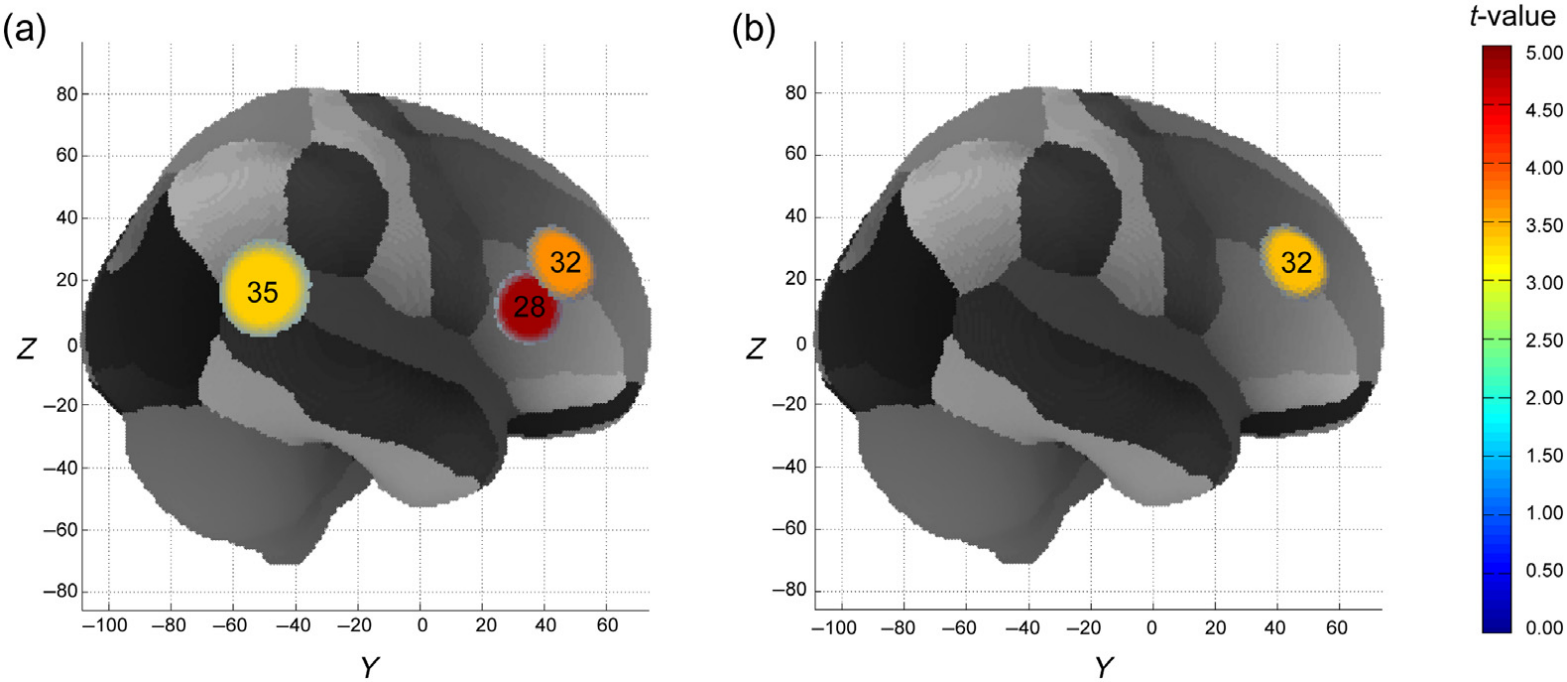
Channels with significant differences between groups detected by t-test with the $M_{\mathrm{eff}}$ method for each experimental dataset: (a) Monden et al. (b) Nagashima et al. Circles indicate significant channels and their locations in MNI space. The numbers in the circles represent channel numbers. The colors of the circles represent t-values based on an independent two-sample t-test (see color bar to the right).

**Fig. 7:**
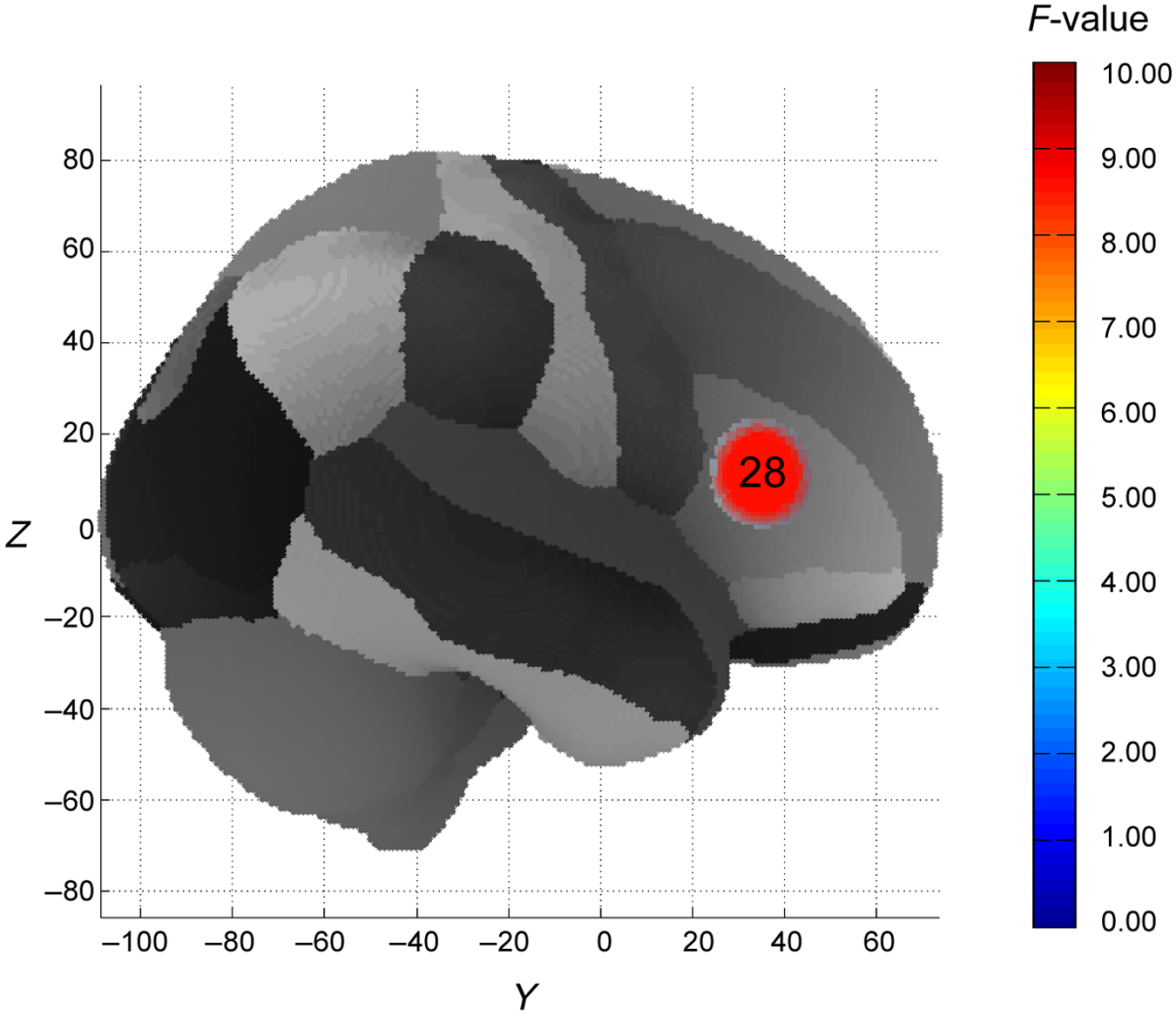
Channels with significant differences among TD, ADHD without ASD, and ADHD with ASD groups detected by one-way randomized-groups ANOVA with the $M_{\mathrm{eff}}$ method. The circle indicates the significant channel and its location in the MNI space. The number in the circle represents the channel number. The color of the circle represents its F-value (see color bar to the right).

For the Monden et al.^22^ dataset, an independent two-sample t-tests with $M_{\mathrm{eff}}$ correction detected significant differences between the TD and ADHD groups at CH28, CH32, and CH35 (Fig. 4). Significant differences between groups at CH28 and CH32, defined as an ROI in previous studies, were successfully reproduced, and an additional significant difference was observed at CH35. For the Nagashima et al.^21^ dataset, a significant difference at CH32 was detected using the $M_{\mathrm{eff}}$ method (Fig. 6). The previous study had identified CH32 as the sole ROI and reported a significant difference between groups. Thus, the results were successfully reproduced. Using experimental data from Monden et al.^22^ and Tokuda et al.,^23^ a one-way randomized-groups ANOVA with the $M_{\mathrm{eff}}$ method detected a significant difference only at CH28 (Fig. 7). Post-hoc pairwise comparisons using independent two-sample t-tests with Bonferroni correction revealed a significant difference between the TD group and the ADHD without ASD group. In the uncorrected pairwise comparisons, significant differences were observed between the TD group and both the ADHD ADHD without the ASD group and the ADHD with ASD group. The significant difference detected by the one-way randomized-group ANOVA with the $M_{\mathrm{eff}}$ method reflects the post hoc results between the TD and both ADHD groups, reproducing the results of Monden et al.^22^

## 4 Discussion and Conclusion

### 4.1 Overview

The aim of this study was to evaluate the applicability of the $M_{\mathrm{eff}}$ method in independent or randomized-group experimental designs and discuss approaches for addressing the multiple testing problem in fNIRS CH-wise analysis. Resampling simulations and the exploratory analyses using actual experimental data, with the number of total participants exceeding the number of CHs, demonstrated that the $M_{\mathrm{eff}}$ correction method provides higher statistical power compared with the Bonferroni correction while maintaining better control of false positives than the FDR method and no correction. This result arises from its ability to account for inter-channel correlations through an eigenvalue decomposition approach, which helps maintain a balance between type I and type II errors. Its lower impact on statistical power compared with other FWER correction methods, even as the number of CH increases, makes it particularly useful for exploratory analyses. Specifically, although conventional FWER approaches typically require the number of tested CHs or ROIs to be restricted to preserve statistical power, the $M_{\mathrm{eff}}$ method appeared to maintain statistical power even when all CHs were tested. This finding also suggests that the $M_{\mathrm{eff}}$ method, which was previously limited to one sample or paired t-tests, can be extended to independent-samples t-tests and one-way randomized-groups ANOVA. Given this advantage, the method continues to be a promising approach for fNIRS analysis with improved spatial resolution through multichannelization. A further expansion of its applicability in fNIRS studies is expected.

### 4.2 Interpretation of Results

#### 4.2.1 Evaluation of $M_{\mathrm{eff}}$ Using Simulated Data

As shown in Fig. 3, the relationship between the total number of CHs tested and the number of significant CHs illustrates the characteristics of each correction method in terms of controlling false positives and maintaining statistical power. The number of false positives, where the number of significant CHs exceeded the number of TS CHs, increased monotonically when a multiple-comparison correction method was not applied. This result suggests the importance of applying a multiple-comparison correction method because false positives increase as the total number of CHs increases. Even when the FDR method was applied, CHs corresponding to false positives also increased. As the FDR method controls the overall error rate at the group level, it does not guarantee the reliability of each test. Therefore, although it is useful in exploratory analyses for detecting significant results and focusing hypotheses, its use in confirmatory analyses is inappropriate. When the Bonferroni correction was applied, no false positives were detected, but as the total number of CHs increased, the number of detected significant CHs fell below the number of truly significant CHs. This is because the Bonferroni correction assumes that all CHs are independent to control the FWER, increasing the risk of false negatives in CH-wise analysis.

Both the $M_{\mathrm{eff}}$ method and the permutation method similarly demonstrated decreases in the number of detected significant CHs as the total number of CHs increased, but these decreases were smaller than those observed with Bonferroni correction. Similar to the $M_{\mathrm{eff}}$ method, the permutation method effectively controlled false positives with a relatively small variation in the number of significant CHs. However, particularly in the one-way randomized-groups ANOVA, a reduction in statistical power with the permutation method was observed compared with the $M_{\mathrm{eff}}$ method (Fig. 4). This decline is likely due to the probability that the max T and max F distributions are more likely to include spuriously significant CHs as the number of CHs increases, which reduces statistical power. On the other hand, the $M_{\mathrm{eff}}$ method also successfully controlled for false positives, and minimal reductions in statistical power were observed compared with the other FWER correction methods, even as the total number of CHs increased (Figs. 3 and 4). As the $M_{\mathrm{eff}}$ value is derived from the overall inter-CH correlations and is used to adjust the α-level for each test, it is robust to variations in the number of CHs. Therefore, the $M_{\mathrm{eff}}$ method can be considered a useful FWER correction method for both exploratory and confirmatory analyses in channel-wise analysis.

#### 4.2.2 Application to Actual Experimental Data

In the resampling simulations (data shown in Fig. 5), $M_{\mathrm{eff}}$ values increased monotonically with an increasing number of CHs, but the increase was not linear. The $M_{\mathrm{eff}}$ values converged to around 25 in the simulated datasets of two and three independent groups. As demonstrated in previous studies,^9^^,^^18^ the $M_{\mathrm{eff}}$ approach effectively suppressed the inflation of multiplicity, considering the correlation between CHs. In addition, the variation in $M_{\mathrm{eff}}$ values across datasets indicates that a data-driven approach has been successfully implemented, accounting for the fNIRS data structure.

The results of applying the $M_{\mathrm{eff}}$ method to independent experimental data were generally consistent with those of the previous studies.^21^^–^^23^ Although each previous study set ROIs, this study performed exploratory statistical analysis across all CHs and detected significant differences in CHs similar to those identified in previous studies. These findings suggest that the $M_{\mathrm{eff}}$ correction method is also effective for conducting exploratory research in independent designs.

### 4.3 Implications

The results of this study provide important guideposts for the future implementation of fNIRS channel-wise analysis. First, correction methods that do not account for the correlations between CHs, which are characteristic of fNIRS data structure, involve the risk of false positives and negatives. In fNIRS studies, the risk of false positives and negatives and the issue of reproducibility that varies with analytical pipelines have been pointed out.^25^^,^^26^ To deal with this issue, it is necessary to select multiple-comparison correction methods that are aligned with the fNIRS data structure. Alternatively, analyses should be divided into exploratory and confirmatory processes.^11^ The conservative Bonferroni correction is recommended only when the ROI is limited to a single CH or a small number of CHs, based on previous studies or preliminary experiments. The FDR method should be employed for exploratory analyses at the initial phase, whereas other FWER correction methods should be applied at the subsequent confirmatory stage.

In addition, this study demonstrated that the $M_{\mathrm{eff}}$ method is effective in balancing type I and type II errors in independent designs and that it is applicable to both exploratory and confirmatory analyses. In fact, the $M_{\mathrm{eff}}$ method has already been applied in genetic and psychological studies using datasets with independent designs.^10^^,^^11^^,^^13^ The results of the resampling simulations in this study suggest that its applicability can also be extended to independent designs in multichannel fNIRS analyses. Although this method has been used in one-sample schemes to determine whether each CH is active, it can also be utilized in experimental designs with different subject groups, such as clinical studies. Moreover, the $M_{\mathrm{eff}}$ value is calculated from correlation coefficients between CHs and, thus, does not require the high computational costs necessitated in a resampling-based approach. The $M_{\mathrm{eff}}$ method was able to produce results equal to or competitive with those of permutation tests, which require a number of iterations to improve accuracy. However, as implied by the results of resampling simulation, when the number of CHs is large, the possibility of false negatives may remain, even when the $M_{\mathrm{eff}}$ method is applied. Therefore, it is still preferable to define ROIs *a priori* whenever possible, even when using the $M_{\mathrm{eff}}$ method.

### 4.4 Limitations and Future Prospects

One of the important considerations with the $M_{\mathrm{eff}}$ method is the sample size. When the sample size, defined here as the number of participants, is smaller than the number of variables, defined here as the number of CHs, rank deficiency can occur in the eigenvalue vector and correlation matrices. When the number of participants is smaller than the number of CHs, the rank of the correlation matrix and eigenvalue vector equals the number of participants minus one. Hence, in such a situation, the $M_{\mathrm{eff}}$ value will be underestimated because not all eigenvalues are calculated. In this study, the eigenvalue rank deficiency issue was not significant because even though the number of participants from one group was smaller than the number of CHs, the number of participants could be supplemented by drawing from two or three independent groups. Specifically, the number of participants exceeded the number of CHs in both datasets, with 60 participants in the t-test dataset and 62 participants in the ANOVA dataset, compared with 44 CHs. Furthermore, even if the sample size is smaller than the number of participants, if the sample size is ∼60% or more of the number of CHs, a valid $M_{\mathrm{eff}}$ can be estimated using a typical exponential model.^18^ Thus, using the current 44 CHs as an example, a sample of ∼27 participants in total would make the $M_{\mathrm{eff}}$ method at least applicable. This number of participants should be feasible, considering ∼14 participants per group in two independent groups and approximately nine participants per group in three independent groups when using 44-CH fNIRS measurement. However, current fNIRS machines allow measurements of more than 44 CHs, making it difficult to satisfy the minimum number of participants necessary for applying the $M_{\mathrm{eff}}$ method. Furthermore, although the current analysis was based on channel-wise data, analysis based on 2D or 3D reconstructed data has become increasingly popular,^27^^–^^32^ further expanding the number of variables, which in this case corresponds to the number of pixels or voxels. Thus, the application of the $M_{\mathrm{eff}}$ method to datasets where the number of participants is much smaller than the number of variables should be considered, and this is further discussed in the next paragraph. However, in current practice, when applying the $M_{\mathrm{eff}}$ method, it is necessary to first confirm whether the sample size of the dataset satisfies the methodological requirements, namely, whether the total number of participants exceeds 60% of the number of CHs or not.

In the current study, we limited our approach to channel-wise analysis, which assumes that the number of variables is smaller than the number of total participants. However, as stated above, there are situations where the opposite becomes likely, such as analysis using 2D or 3D reconstructed images. In such imaging data, the variables being tested correspond to the number of pixels or voxels, far exceeding the number of total participants and even 60% of the number of total participants,^18^ which is the minimum value necessary for use of $M_{\mathrm{eff}}$ correction. Therefore, the conventional Galwey formula becomes challenging to apply, as the rank deficiency of eigenvalues becomes a critical issue. To address the problem of rank deficiency, Moskvina and Schmidt^15^ and Chen and Liu^17^ proposed a fixed formula for calculating $M_{\mathrm{eff}}$ directly from the correlation coefficient matrix. This allows calculating $M_{\mathrm{eff}}$ without eigenvalue decomposition, which could avoid the potential issue of rank deficiency of eigenvalues. The applicability of these approaches to pixel- or voxel-wise fNIRS analysis, where the sample size is significantly smaller than the number of variables, represents an intriguing avenue for future research.

## Supplementary Material

10.1117/1.NPh.13.3.035005.s01

## Data Availability

The code needed to conduct this analysis is accessible in our GitHub repository at https://github.com/Dan-brain-Lab. The group-level data used in this study are available to researchers from the corresponding author upon request. Requests should be directed to dan@brain-lab.jp.
