# Supplementary material for: Extending effective multiplicity for independent designs in multichannel fNIRS studies
Source: Neurophotonics. 2026 Aug 12;13(3):035005. doi: 10.1117/1.NPh.13.3.035005 (PMC13464491; doi:10.1117/1.NPh.13.3.035005)
Supplement: Supplementary file 1 [file NPh_013_035005_SD001.docx]

Supplementary Materials

Supplementary Table 1 Average *M*_eff_ values and *SD* of independent *t*-tests and one-way randomized-groups ANOVAs.

| number of channels | independent *t*-test | |  |  | ANOVA | |
| --- | --- | --- | --- | --- | --- | --- |
|  | Average | *SD* |  |  | Average | *SD* |
| 1 | 1.00 | 0.00 |  |  | 1.00 | 0.00 |
| 2 | 1.95 | 0.06 |  |  | 1.97 | 0.05 |
| 3 | 2.85 | 0.10 |  |  | 2.90 | 0.08 |
| 4 | 3.74 | 0.13 |  |  | 3.81 | 0.11 |
| 5 | 4.56 | 0.16 |  |  | 4.68 | 0.13 |
| 6 | 5.38 | 0.19 |  |  | 5.52 | 0.15 |
| 7 | 6.16 | 0.20 |  |  | 6.36 | 0.18 |
| 8 | 6.90 | 0.23 |  |  | 7.16 | 0.19 |
| 9 | 7.65 | 0.24 |  |  | 7.93 | 0.21 |
| 10 | 8.34 | 0.25 |  |  | 8.70 | 0.23 |
| 11 | 9.03 | 0.28 |  |  | 9.42 | 0.24 |
| 12 | 9.69 | 0.30 |  |  | 10.15 | 0.25 |
| 13 | 10.34 | 0.29 |  |  | 10.86 | 0.26 |
| 14 | 10.96 | 0.31 |  |  | 11.56 | 0.27 |
| 15 | 11.56 | 0.32 |  |  | 12.22 | 0.28 |
| 16 | 12.17 | 0.32 |  |  | 12.85 | 0.28 |
| 17 | 12.74 | 0.33 |  |  | 13.50 | 0.29 |
| 18 | 13.30 | 0.32 |  |  | 14.13 | 0.30 |
| 19 | 13.82 | 0.34 |  |  | 14.72 | 0.31 |
| 20 | 14.37 | 0.35 |  |  | 15.34 | 0.31 |
| 21 | 14.90 | 0.34 |  |  | 15.92 | 0.31 |
| 22 | 15.40 | 0.34 |  |  | 16.49 | 0.31 |
| 23 | 15.91 | 0.33 |  |  | 17.05 | 0.32 |
| 24 | 16.40 | 0.33 |  |  | 17.61 | 0.31 |
| 25 | 16.84 | 0.33 |  |  | 18.15 | 0.32 |
| 26 | 17.29 | 0.33 |  |  | 18.69 | 0.30 |
| 27 | 17.75 | 0.33 |  |  | 19.18 | 0.31 |
| 28 | 18.20 | 0.32 |  |  | 19.68 | 0.30 |
| 29 | 18.63 | 0.31 |  |  | 20.17 | 0.30 |
| 30 | 19.04 | 0.31 |  |  | 20.67 | 0.30 |
| 31 | 19.45 | 0.30 |  |  | 21.14 | 0.27 |
| 32 | 19.84 | 0.29 |  |  | 21.60 | 0.27 |
| 33 | 20.23 | 0.28 |  |  | 22.06 | 0.26 |
| 34 | 20.61 | 0.27 |  |  | 22.49 | 0.26 |
| 35 | 20.97 | 0.26 |  |  | 22.93 | 0.24 |
| 36 | 21.34 | 0.25 |  |  | 23.36 | 0.23 |
| 37 | 21.69 | 0.23 |  |  | 23.77 | 0.21 |
| 38 | 22.04 | 0.22 |  |  | 24.19 | 0.20 |
| 39 | 22.38 | 0.20 |  |  | 24.59 | 0.19 |
| 40 | 22.70 | 0.18 |  |  | 24.99 | 0.18 |
| 41 | 23.03 | 0.16 |  |  | 25.37 | 0.15 |
| 42 | 23.33 | 0.13 |  |  | 25.75 | 0.13 |
| 43 | 23.64 | 0.10 |  |  | 26.11 | 0.09 |
| 44 | 23.94 | 0.00 |  |  | 26.47 | 0.00 |
